# Does undertaking an intercalated BSc influence first clinical year exam results at a London medical school?

**DOI:** 10.1186/1472-6920-11-6

**Published:** 2011-02-03

**Authors:** Mary Howman, Melvyn Jones

**Affiliations:** 1 Department of Primary Care and Population Health, UCL Medical School, London, UK

## Abstract

**Background:**

Intercalated BScs (iBScs) are an optional part of the medical school curriculum in many Universities. Does undertaking an iBSc influence subsequent student performance? Previous studies addressing this question have been flawed by iBSc students being highly selected. This study looks at data from medical students where there is a compulsory iBSc for non-graduates. Our aim was to see whether there was any difference in performance between students who took an iBSc before or after their third year (first clinical year) exams.

**Methods:**

A multivariable analysis was performed to compare the third year results of students at one London medical school who had or had not completed their iBSc by the start of this year (n = 276). A general linear model was applied to adjust for differences between the two groups in terms of potential confounders (age, sex, nationality and baseline performance).

**Results:**

The results of third year summative exams for 276 students were analysed (184 students with an iBSc and 92 without). Unadjusted analysis showed students who took an iBSc before their third year achieved significantly higher end of year marks than those who did not with a mean score difference of 4.4 (0.9 to 7.9 95% CI, p = 0.01). (overall mean score 238.4 "completed iBSc" students versus 234.0 "not completed", range 145.2 - 272.3 out of 300).

There was however a significant difference between the two groups in their prior second year exam marks with those choosing to intercalate before their third year having higher marks. Adjusting for this, the difference in overall exam scores was no longer significant with a mean score difference of 1.4 (-4.9 to +7.7 95% CI, p = 0.66). (overall mean score 238.0 " completed iBSc" students versus 236.5 "not completed").

**Conclusions:**

Once possible confounders are controlled for (age, sex, previous academic performance) undertaking an iBSc does not influence third year exam results. One explanation for this confounding in unadjusted results is that students who do better in their second year exams are more likely to take an iBSc before their third year.

## Background

A BSc, BMedSci or BA degree (iBSc) has long been an optional part of a medical degree in the United Kingdom. Students choose to undertake an extra year of study during their five year medical degree and this is known as intercalating. This contrasts with the practice in some other countries such as the United States where all students are graduates prior to starting a medical degree. In "Tomorrow's Doctors" the UK General Medical Council (GMC) stresses the importance of student-selected components but does not mention BScs specifically as it did in the first edition. However, an intercalated BSc (iBSc) could be described as an example of a student-selected component (SSC). The recently published 2009 edition of "Tomorrow's Doctors" states, "The purpose of SSCs is the intellectual development of students through exploring in depth a subject of their choice." Historically in the UK around a third of students have chosen to take up the option of an intercalated degree [1].

Many factors may feed into a student's decision about intercalating. Agha found that the most common reason for students choosing to do an intercalated degree was that they felt it would improve their career prospects [2]. There is certainly evidence that this is the case if they wish to pursue academic careers; Evered showed that professors and readers are more likely to have an intercalated degree and those academics with such a degree were more likely to obtain research grants [3]. It is not clear from those studies whether this is due to selection bias with more academically-minded students taking iBScs, or whether the iBSc itself provides skills and knowledge that lead to better performance. However, a longitudinal study by McManus suggested the latter, that students who intercalated developed deeper analytical skills and strategic learning, although this effect was diluted with increasing numbers of intercalating students [1].

Another consideration for students deciding whether to intercalate is the application process for their first jobs. When students apply for their first jobs as doctors (foundation year posts), they are given points based on standardised academic and non-academic criteria. The number of points they have compared to others determines the likelihood of them getting the job of their choice. Intercalating gains points for students in the ranking system for allocating students to foundation year posts, making it more likely they will get their preferred post [4].

Potential disadvantages of undertaking an intercalated degree are the extra cost of over £40,000 in funding via SIFT payments (medical service increment for teaching), and obviously there will be expense and possible increased debt for the student, an extra year spent at medical school, and possible difficulty in adjusting to clinical studies [5]. Students may feel an iBSc is for the more academically orientated and so not for them. It also delays entry of junior doctors into the NHS workforce for one year.

Is there any evidence that undertaking an iBSc improves subsequent clinical exam results? This may be key when students who feel they do not want to pursue an academic career are considering whether or not to intercalate. Perhaps if this could be proven more students would take up the option and so reap the subsequent benefits of exposure to an academic environment. Equally it may be of interest to medical schools considering whether or not to make iBScs a compulsory part of the course.

There have been some attempts to address this question. The results so far are conflicting. A small study by Tait at Birmingham showed no significant effect on clinical examination results of having an iBSc [6]. However, a study looking at the Pathology iBSc in Edinburgh found that those who had undertaken this did subsequently obtain better clinical exam results [7]. A more recent study at Aberdeen, which adjusted for confounders such as prior performance, looked at 891 students of which 18% had done an intercalated degree. They found that these students did better in three out of six subsequent clinical exams, including the written exam and Objective Clinical Structured Exam (OSCE) [4].

Selection bias has always hindered attempts to study the impact of iBScs, as students offered an iBSc have been selected on prior academic performance. Crucially, UCL's compulsory introduction of an iBSc allows us to examine this question with a reduced risk of selection bias.

UCL students can choose from degrees offered by twenty one departments including anatomy and developmental biology, biochemistry and molecular biology, child health, history of medicine, genetics, immunology, infection, clinical sciences international health, medical anthropology, medical physics, molecular medicine, neuroscience, orthopaedic science, pharmacology, philosophy, physiology, primary care, psychology, speech science and surgical sciences. The majority of these iBScs can be taken after their second, third or fourth year and students will apply for their iBSc at their chosen time; the clinical sciences iBSc cannot be taken after the second year. If a course is oversubscribed candidates' applications including a reference, evidence of academic ability and personal statement are considered on merit.

These courses consist of subject specific elements, development of core skills such as critical appraisal and research methods and an element of research such as a library project, laboratory based experiment or a clinical study. UCL's student guide to iBScs states ^"^we believe students obtain considerable benefit from the intellectual experience of pursuing knowledge for its own sake, learning very valuable transferable skills not least in critical thinking and evaluation of evidence." So, the aim of an iBSc is wider than improving knowledge in one particular field; rather it also aims to improve students' professional skills such as problem solving and assessing evidence.

The aim of this study was therefore to examine the impact of iBScs (in a medical school where they are compulsory for non-graduates) by looking to see if there was any difference in performance between students who took an iBSc before or after the academic year 2005/6 by comparing third year exam results from this year.

## Methods

iBScs became compulsory in the UCL medical school from the student intake in 2000. The core medical MBBS course at UCL is five years and split in to three phases; Phase One "Science and Medicine" covers basic sciences during the first and second years, Phase 2 "Science and Medical Practice" introduces the students to clinical skills in their third and fourth years and Phase 3 "Preparation for Practice" consolidates their skills in the fifth year. As described, students can intercalate after their second, third or fourth year making the entire course six years long.

**Figure 1:**
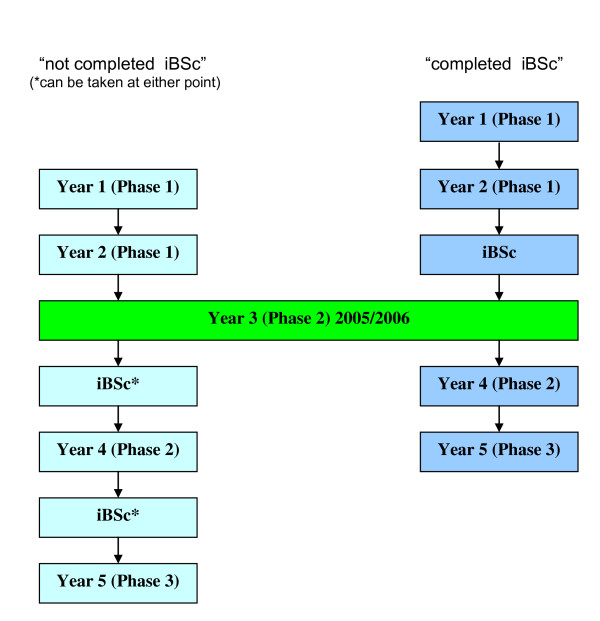
**Showing the two groups of UCL medical students being compared, those who took their iBSc after their third year ("not completed iBSc") on the left, and those who took it before their third year ("completed iBSc") on the right**.

This compulsory introduction of iBScs provided an opportunity to compare clinical exam results in students who had and had not taken an iBSc by looking at results from a clinical exam mid-way through the course. Figure 1 shows the progress of students through the medical course at UCL and the two groups of students being compared: "completed iBSc" students who had already done their iBSc when they sat their third year exams and "not completed iBSc" students who would do their iBSc after their third year.

The outcome measures were summative assessments used at the end of year three (previously called first clinical year). They consisted of an overall end of year result and three component parts: Objective Structured Clinical Exam (OSCE), a written exam and logbook mark. The OSCE examined students' ability to demonstrate a variety of different tasks including history taking, examination skills, data interpretation and clinical skills such as catheterisation. The written paper was a mixture of multiple choice questions and extended matching questions and the log book was scored on a combination of subjective clinical firm grades, work based assessments such as case based discussions and clinical examinations performed during hospital attachments. The OSCE and written exams had a Kuder-Richardson Formula 20 (KR-20) score for estimating test reliability of over 0.7 suggesting they reliably distinguished between two groups of students [8].

Study data was sourced from the medical school student records system and de-identified to ensure student anonymity. As well as third year exam results, data included age, gender, nationality and baseline performance. The latter was measured in two ways; by three highest A level results achieved by the student (where A grade = 3 points, B = 2, C = 1) and also by second year exam results.

A retrospective observational study was undertaken based on anonymised electronic records of students in their third year 2005/6. Graduates (degree holders on admission to medical school) and transfers (from other universities) were excluded from the analysis because they already had degrees and were not eligible to take an iBSc as part of the medical course. Other students identified as being exempt from an iBSc were also excluded; exemptions were agreed on an individual basis by the medical school based on financial and personal circumstances with the majority of exemptions being for overseas students with financial difficulties, although many overseas students do intercalate.

Baseline statistical analysis was undertaken with Pearson Chi squared tests used for categorical values and an independent samples t-test for non-categorical values using SPSS version 15.0. A multivariable analysis was performed on overall exam results and component parts (OSCE, written exam, logbook). The result was then adjusted using a general linear model for the effects of age, gender, nationality (whether students were home or overseas students) and baseline performance (A levels and year two score). The level of statistical significance was set at alpha = 0.05.

Ethical approval was discussed with the UCL ethics committee and it was deemed not to be required. Permission was gained from the medical school. Ethical principals were adhered to in this research project; data was anonymised and registered with UCL data protection.

## Results

**Figure 2:**
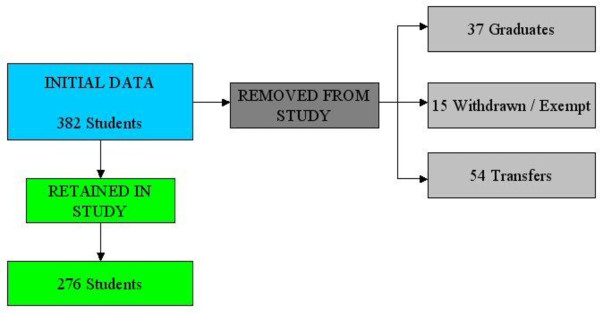
**Flow chart of study recruitment of students**.

We collected data on 382 students of which 276 were eligible for analysis. Predominantly students were UK nationals, but 38 were non-British nationals of which 9 were European Union students. Figure 2 shows the student numbers involved in the final analysis. The results showed 184 students had completed the BSc (66.7%) and 92 had not completed one (33.3%).

There were some baseline differences between the groups as highlighted in Table 1, with those not having completed an iBSc being younger as they had not taken an extra year to do the iBSc. There was no significant difference in A level grades but there was a significant difference between the two groups in their second year exam score, with those having completed an iBSc having higher scores.

**Table 1:** Baseline differences between students who had completed and those who had not completed their iBSc.

	Completed iBSc Group	Not completed iBSc Group	Statistical significance
Female	99 (54%)	59 (64%)	p = 0.10

Male	85 (46%)	33 (36%)	p = 0.10

Home student	163 (89%)	75 (82%)	p = 0.11

Overseas student	21 (11%)	17 (18%)	p = 0.11

Average Age (years)	21.98	21.22	**p < 0.001**

Average A level score (A = 3, B = 2, C = 1)	8.52	8.34	p = 0.12

Score (%) in second year exam, range 38.4-86.4	66.03	58.56	**p < 0.001**

Statistically significant results shown in bold.

There was a significant mean difference in end of third year exam scores of 4.4 (0.9 - 7.9 95% confidence interval, p = 0.01). The group that had completed an iBSc scored higher (238.4 "completed iBSc" students versus 234.0 "not completed ", score range 145.2 to 272.3 out of 300).

Looking at the individual exam components, students who had completed an iBSc did significantly better in the written paper (mean score of 76.4/100 "completed" compared to 72.7 "not completed", mean difference 3.7, 2.2 - 5.1 95% confidence interval, p < 0.001). The completed iBSc group also got higher logbook scores with a mean score of 85.4/100 compared to 83.3 in the not completed iBSc group (mean difference 2.1, 0.6 - 3.6 95% confidence interval, p = 0.01). There was no significant difference between the two groups when it came to OSCE scores with a trend towards the group having completed an iBSc scoring less well, 76.6 out of 100, with those not having completed an iBSc scoring 78.0 (mean difference -1.4, -3.0 - 0.2 95% confidence interval, p = 0.10). These results are summarized in Table 2.

**Table 2:** The difference in exam grades between the completed and not completed iBSc students (to one decimal place).

	Completed iBSc group	Not completed iBSc group	Mean score difference	Statistical significance	Completed iBSc group adjusted score	Not completed iBSc group adjusted score	Statistical significance of difference in adjusted scores
Overall score	238.4	234.0	4.4 (95% CI 0.9-7.9)	**p = 0.01**	238.0	236.5	NS

Written paper	76.4	72.7	3.7 (95% CI 2.2-5.1)	**p < 0.001**	76.1	74.9	NS

Log Book	85.4	83.3	2.1 (95% CI 0.6-3.6)	**p = 0.01**	85.2	84.8	NS

OSCE	76.6	78.0	1.4 (95% CI -3.0-0.2)	p = 0.10	76.7	76.9	NS

NS = not significant.

Adjusting for age, gender, nationality and baseline A level performance, the difference in mean overall score between the completed and not completed iBSc groups was still significant (p = 0.01). However, after further adjusting the model for the difference in year two exam grades, the difference between the completed and not completed iBSc groups was no longer significant (difference in mean adjusted score 1.4, -4.9 to +7.7 95% confidence interval, p = 0.66). The mean adjusted score for those who had completed an iBSc was 238.0 and those who had not was 236.5 (rounded to one decimal place). Second year exam scores had a significant effect on third year exam scores in the general linear model (p < 0.001).

## Discussion

This study looks at the effect of undertaking an iBSc on subsequent exam performance in a medical school where iBScs are compulsory, the first study where this is the case and where selection bias is reduced in this way.

Our findings suggest that students who have done iBScs do not do better or worse in subsequent clinical exams. Although the unadjusted analysis suggests an improvement in results following an iBSc, once prior performance in second year exams is taken into account there appears to be no benefit or detriment from intercalating when considering subsequent exam results. It appears that what is happening is that the students who are achieving better exam results in their second year are more likely to choose to complete their iBSc before their third year (first clinical year) and this difference in baseline performance may be what leads to the unadjusted difference in third year exam results, rather than the effect of the iBSc per se. Those achieving better second year exam results may be more likely to complete their iBSc early (before their third year), as there is competition for iBScs with those with better second year results being more likely to gain early places on the iBSc of their choice. Those with worse results may not get the iBSc of their choice and so choose to do a later iBSc or they may simply "put off" doing an iBSc for as long as possible. Having said this some iBScs, for example those in clinical sciences, can only be undertaken after the third year so other variables are also involved in determining when a student decides to intercalate, for example their preferred subject choice. The results may also reflect McManus' finding that the improvement in deep learning is diluted the more students undertake an iBSc [1]; this may be as students are no longer self selected or be because resources are stretched.

This result could be of interest to medical schools considering making intercalation compulsory, or thinking about when to offer iBScs and how many to offer. It may be reassuring to medical schools and students, allaying concerns that a year spent doing an iBSc away from the core curricula might be detrimental to subsequent clinical results. Equally it may be of concern; in times where resources are tight, medical schools and students could feel they want an extra year spent studying to have concrete benefits such as an improvement in clinical exam results and therefore ranking for foundation year jobs. It may therefore provide evidence that could be used to argue against compulsory iBScs as part of a medical degree. However, this study measures the impact of an iBSc purely on subsequent clinical exam results; obviously there are likely to be many other benefits of taking an iBSc, for example development of research skills, which are not accounted for in this study but are likely to be factors taken into account by medical schools and students when considering the value of iBScs.

The result of this study may be generalisable to other medical schools offering compulsory iBScs though there will be differences in the disciplines and content of iBScs offered at different institutions. It would be interesting to look at the type of iBSc taken, for example whether it is science or humanities based, and see if this has an effect on subsequent clinical exam results. The results of this study may not be generalisable to schools offering fewer iBScs because of the dilution effect described by McManus [1].

There are limitations to the study. It relies on the use of routine data collected about students and there may be inherent inaccuracies in this. There may be other explanations or confounders that influence students' choice about iBSc timing and therefore influence our results, such as the psychological profile (stress, personal difficulties) or the socio-demographic profile (parental support or debt) of students, but we are unable to comment on this with our data set.

## Conclusion

This study set out to see if iBScs impact on undergraduate exam performance, in a setting where selection bias as an issue had been reduced, in a school where an iBSc is compulsory. Despite this design the results still appeared to be affected by selection bias. A randomised controlled trial would eliminate such bias but would be impracticable and probably unethical as students cannot be forced to take a particular iBSc at a particular time, and so is not a likely option for further study.

Further quantitative research would be useful, perhaps concentrating on the type of iBSc undertaken and whether this influences subsequent results. Qualitative research may help elicit whether there are other potential unrecognised confounders between the groups choosing to undertake their iBSc at different times and explore the decision-making process around iBScs. Through in-depth interviews with students who have decided whether and when to intercalate, the impact of psychological and socio-economic factors on this decision can be explored as well as factors that have not been previously considered.

There is a definite need for further research in this area, which is sparse at present, so that both medical schools and students can make informed decisions about intercalating.

## Competing interests

Non-financial competing interests. MJ was a course organiser for an iBSc in primary health care until 2005. MJ & MH are both student project supervisors for an iBSc.

## Authors' contributions

MH collected and analysed the data and drafted the paper. MJ conceived the original idea and was involved throughout helping with data analysis and critically revising the written submission.

## Authors' information

Dr Mary Howman is a GP in London and Clinical Teaching Fellow at UCL.

Dr Melvyn Jones is a GP and Senior Lecturer at UCL where he ran a BSc in primary health care. He now runs the year 3 community based teaching programme. He is a GP in Hertfordshire.

## Pre-publication history

The pre-publication history for this paper can be accessed here:

http://www.biomedcentral.com/1472-6920/11/6/prepub
